# A Rare Case of Penile Metastases as a Harbinger of Primary Pulmonary Adenosquamous Carcinoma

**DOI:** 10.1155/2018/8361368

**Published:** 2018-03-26

**Authors:** Partha Hota, Tejas N. Patel, Xiaofeng Zhao, Carrie Schneider, Omar Agosto

**Affiliations:** ^1^Division of Abdominal Imaging, Department of Radiology, Temple University Hospital, Philadelphia, PA, USA; ^2^Division of Abdominal Imaging, Atlantic Medical Imaging, Galloway, NJ, USA; ^3^Department of Pathology, Temple University Hospital, Philadelphia, PA, USA

## Abstract

Although lung cancer has a high propensity for distant metastatic disease, penile metastases from primary lung neoplasms are considered particularly rare. A 71-year-old male presented to our hospital with a rapidly enlarging hard palpable penile mass. MR imaging demonstrated two penile masses centered in the left and right corpus cavernosa. Subsequent CT imaging revealed a spiculated pulmonary mass in the right upper lobe with PET/CT, MRI, and surgical exploration, demonstrating evidence of metastases to the left adrenal gland, right subscapularis muscle, brain, and small bowel. Tissue sampling of lesions in the small bowel, right subscapularis muscle, and penis demonstrated histopathology consistent with an adenosquamous carcinoma which in combination with the appearance of the right upper lobe mass on PET/CT imaging suggested the patient's lung cancer as the primary lesion. Prior to our case, pulmonary adenosquamous carcinoma metastasizing to the penis has only been reported once in the literature. Herein, we report a rare case of penile metastases as the presenting sign of metastatic pulmonary adenosquamous carcinoma characterized with PET/CT and MR imaging.

## 1. Introduction

Lung cancer is currently the leading cause of cancer mortality in the United States, contributing to approximately 25% of all cancer-related deaths [1]. Early diagnosis of metastatic disease is crucial for staging and treatment planning with common sites of extrathoracic metastases including the adrenal glands, liver, brain, and bone via hematogenous dissemination [2]. Although the penis has an abundant and highly complex vascular and lymphatic supply, metastatic disease to the penis is quite rare with just over 500 reported cases of penile metastases reported to date [3–5]. Of these patients, there have been only a total of 40 cases of penile metastases from primary lung cancer with the following histopathologic incidences: squamous cell carcinoma (63%), adenocarcinoma (18%), and a single reported case of adenosquamous carcinoma (ASC) [3]. Approximately one-third of penile metastases from lung cancer are detected at the time of primary tumor detection with the vast remainder detected several months later, often in end-stage disease [3]. Penile metastases are rarely detected prior to the diagnosis of a primary lung cancer with only four cases reported in the current literature [2, 3, 6, 7]. We report a rare case of penile metastases as the presenting sign of a metastatic ASC of the lung.

## 2. Case Report

A 71-year-old male with a history of hypertension and hyperlipidemia and a 70 pack-year smoking history presented to our hospital with a two-month history of an enlarging penile mass at the base of the shaft as well as a 10-pound weight loss. Physical examination demonstrated a hard, smooth, approximately 2 cm mass surrounding the base of the penile shaft. In addition, both testes were distended. No palpable pelvic lymphadenopathy was found and vital signs and laboratory data were within normal limits.

**Figure 1:**
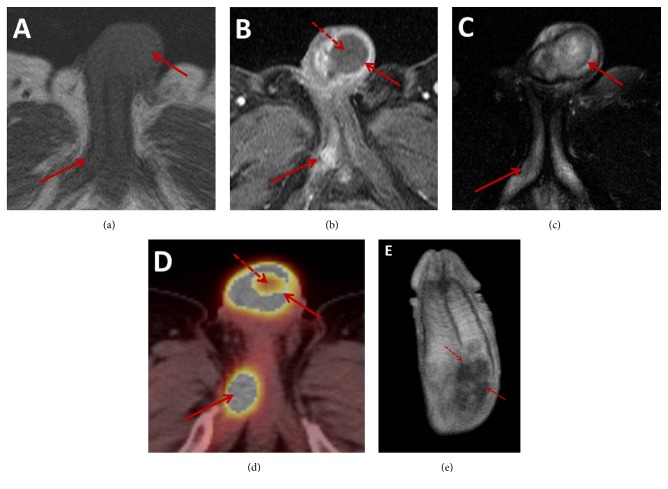
Axial T1-weighted MR images (a) demonstrate two isointense penile masses involving the left paracentral shaft base and proximal right corpus cavernosum (solid arrows) demonstrated with enhancement (solid arrow) on axial contrast enhanced T1-weighted MR images (b). The larger lesion at the base of the shaft demonstrates peripheral enhancement and central nonenhancement (dashed arrow), while the smaller posterior lesion homogeneously enhances. Both lesions demonstrate intrinsic heterogeneous hyperintensity (solid arrow) on axial T2-weighted MR imaging (c). Corresponding to the areas of enhancement on postcontrast MR images, PET/CT imaging (d) demonstrates hypermetabolic activity (solid arrow). A central area of decreased metabolic activity within the larger lesion (dashed arrow) corresponds to the area of central nonenhancement on MRI in keeping with central necrosis. Coronal contrast-enhanced T1-weighted MR image (e) demonstrates the larger lesion (solid arrow) extending across the intercorporal septum (dashed arrow).

**Figure 2:**
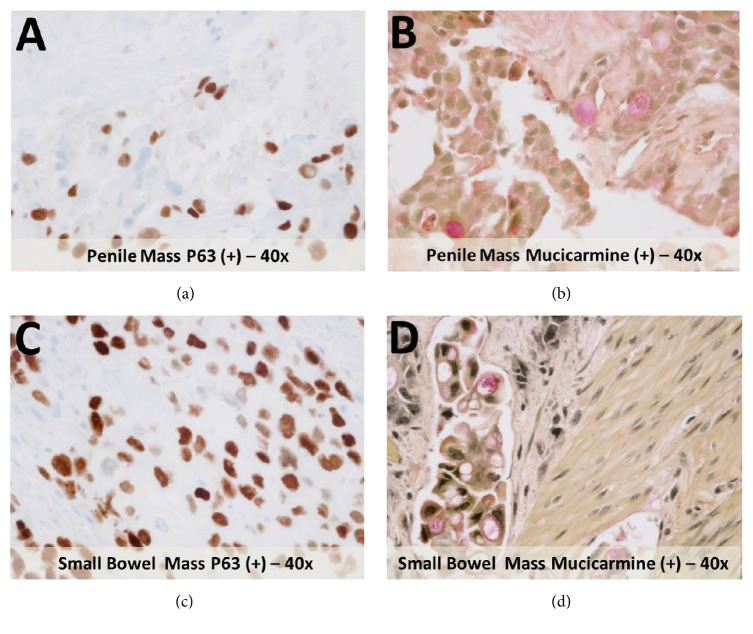
Immunohistochemical analysis of the penile mass demonstrates positive staining with an indicator for squamous cell carcinoma, p63 (a), and positive staining with an indicator for adenocarcinoma, mucicarmine (b). The small bowel mass demonstrates a similar immunostaining profile and is positive with both p63 (c) and mucicarmine (d) immunostains.

Penile magnetic resonance (MR) imaging demonstrated a 6 × 2.5 × 6 cm (AP × TV × CC) irregularly shaped mass centered in the left corpus cavernosum involving the proximal and mid aspects of the penile shaft extending across the intercorporal septum (Figure 1). This lesion demonstrated intrinsic isointensity on T1-weighted images and hyperintensity on T2-weighted images with peripheral enhancement and central hypovascularity. No extension into the corpus spongiosum or penile urethra was identified. A second lesion measuring 3 × 1 cm (AP × TV) demonstrating similar T1 and T2 signal characteristics but with more homogeneous enhancement was present in the more proximal right corpus cavernosum. Transcoporeal biopsy of the larger penile lesion was performed and subsequent immunostaining was positive for squamous cell markers (p63 and CK5/6) as well as an adenocarcinoma marker (mucicarmine) in keeping with an ASC with a predominantly squamous cell pattern (Figures 2(a) and 2(b)) [8].

**Figure 3:**
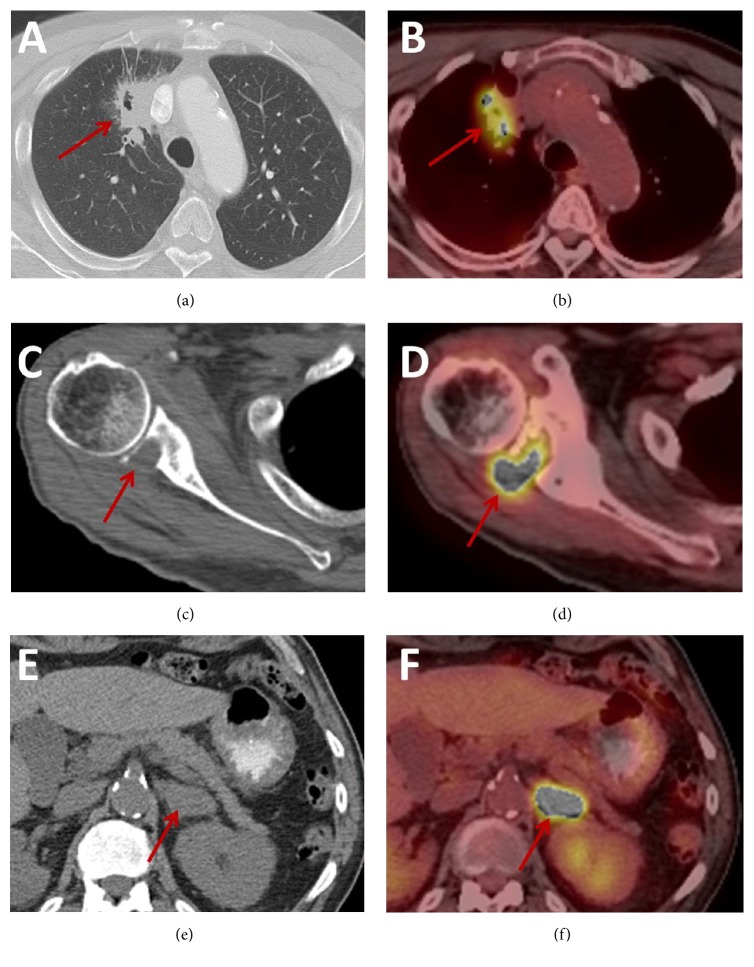
Axial noncontrast CT image in the lung window (a) demonstrates a spiculated mass with an area of central cavitation in the right upper lobe abutting the mediastinum (solid arrow) with imaging features suggesting a primary pulmonary squamous cell carcinoma. The corresponding PET/CT image (b) demonstrates hypermetabolic activity (solid arrow) within this lesion. Axial noncontrast CT image in the bone window (c) demonstrates a soft tissue lesion centered in the right subscapularis muscle with destruction of the adjacent bony glenoid (arrow). Corresponding PET/CT imaging (d) demonstrates hypermetabolic activity (solid arrow) within this lesion. Axial noncontrast CT image in the soft tissue window (e) demonstrates a solid lesion in the left adrenal gland (solid arrow) with the corresponding PET/CT image (f) demonstrating hypermetabolic activity (solid arrow) within this lesion.

**Figure 4:**
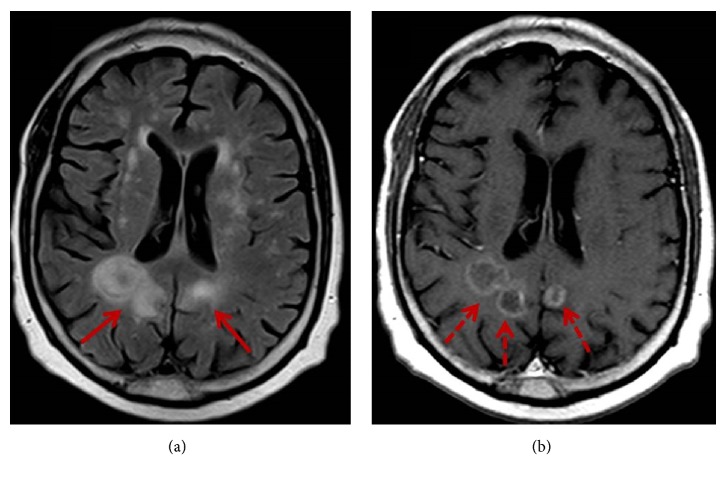
Axial T2-weighted FLAIR (a) and T1-weighted postcontrast MR images (b) demonstrate ring enhancing lesions in the bilateral parietal lobes (dashed arrows) at the level of the basal ganglia with surrounding vasogenic edema (solid arrows) in keeping with brain metastases.

Subsequent whole-body positron emission tomography/computed tomography (PET/CT) imaging demonstrated hypermetabolism of both penile lesions with a maximum standard uptake value (SUV_max_) of 19.9 (Figures 1(d) and 3). In addition, a 4.3 × 2.1 cm (AP × TV) hypermetabolic spiculated pulmonary mass (SUV_max_ = 10.7) was identified in the right upper lobe abutting the mediastinum with areas of central cavitation. The overall imaging features of this lung lesion were suggestive of a primary lung squamous cell carcinoma. Additional mass lesions with similar hypermetabolic activity were identified in the right subscapularis muscle (SUV_max_ = 15) and left adrenal gland (SUV_max_ = 15.3). No hypermetabolic lymphadenopathy was identified. Subsequent contrast-enhanced MR imaging of the brain demonstrated multiple supratentorial and infratentorial ring enhancing lesions in keeping with metastases (Figure 4). Percutaneous biopsy of the right subscapularis mass was performed demonstrating positive immunostaining with p63 and mucicarmine in a pattern similar to the penile masses. Given the similar immunostaining profile of the penile and right subscapularis lesions, the overall distribution of metastases, and appearance of the right upper lobe mass, a unifying diagnosis of stage IV primary ASC of the lung with distant metastases was made.

Six months later, following chemotherapy, the patient returned to our hospital with severe abdominal pain with CT imaging demonstrating a perforated small bowel obstruction with a transition point in the mid-small bowel. The patient underwent explorative laparoscopy and partial small bowel resection. Immunostaining of the resected segment of small bowel demonstrated positive staining with p63 and mucicarmine strongly resembling the staining profile of the penile and subscapularis masses in keeping with metastatic spread to the small bowel (Figures 2(c) and 2(d)). The patient was subsequently discharged from the hospital and died 1 month later from cardiac arrest.

## 3. Discussion

Since secondary penile metastases were first reported by Eberth in 1870, a total of just over 500 cases of this rare entity have been reported in the literature [3, 9]. Of the primary malignancies attributed to penile metastases, approximately 70–75% originate from regional genitourinary or lower gastrointestinal organs of the pelvis [7, 10]. The remainder arise from extrapelvic organs and include tumors of the lungs (4–6.2%), upper gastrointestinal tract, kidneys, hematological system, and osseous structures [3, 4, 7]. Although lung cancer is the second most common malignancy with the estimated 222,500 new cases diagnosed in 2017, secondary penile metastases from lung cancer are particularly rare with only 40 reported cases to date, with our case being the 41st [1, 3, 4, 7].

The mean age of secondary penile metastases is dependent on age of incidence of the primary malignancy and in the setting of the lung cancer is approximately 61 years of age [3, 10]. Approximately one-third of penile metastases from lung cancer are detected at the same time as the primary tumor with the remainder detected in advanced disease at a mean interval of 18 months following primary tumor detection [3, 11]. Penile metastases are rarely detected prior to the diagnosis of the primary lung cancer with only four cases with this presentation reported in the current literature, with our case being the fifth [2, 3, 6, 7].

Clinical manifestations vary widely with the most common symptom being the presence of a palpable penile mass which has been reported in 45% to 80% of patients with an average size of 3.5 cm [3, 5, 6, 10]. Low flow priapism secondary to occlusion of the venous plexus by tumor cells, the so-called “malignant priapism,” has been reported in 20% to 53% of patients with secondary penile metastases and may be a particularly useful clinical tool in differentiating secondary penile metastases from primary penile malignancies as it is almost never observed in the latter [6, 10, 12]. Although penile pain and obstructive uropathy are infrequently reported as initial symptoms, they may be present in advanced disease secondary to increased mass effect and infiltration, notably into the corpus spongiosum [10]. As patients presenting with penile metastases often have widespread metastatic disease, symptoms specific to the primary tumor are common, including dyspnea, cough, and weight loss in the setting of a primary lung malignancy [2, 3, 6].

Distant metastatic disease from primary lung cancer predominantly spreads secondary to hematogenous dissemination of malignant cells via the arterial route [10]. Counterintuitively, despite a robust vascular supply even during flaccidity, extensive vascular communication between the penis and the adjacent pelvis organs, and its location as an end organ of arterial, venous, and lymphatic systems, the overall incidence of secondary penile metastases remains quite low [3, 4]. Several hypotheses have been proposed to explain the pathogenesis behind this enigma including an imperfect penile microenvironment as well as high flow through arterial and venous communications which may lead to difficulty in neoplastic seeding via hematogenous spread [10]. Decreased or retrograde venous flow may facilitate tumor seeding and may explain the relatively high propensity for penile metastases in the setting of neoplasm in neighboring pelvic organs which may cause obstruction of the pudendal plexuses and sluggish or reversed flow [10, 13].

The majority of the reported secondary penile metastases from lung cancer are of squamous cell carcinoma origin (23 of 40 cases) followed by adenocarcinoma origin (7 of 40 cases) [3]. In contrast, the incidence of pulmonary adenocarcinoma is greater than pulmonary squamous cell carcinoma with an incidence of 40% and 25–30% in the lung cancer population, and this discrepancy may be the result of a female-predominant incidence of pulmonary adenocarcinoma [6, 14]. Penile metastasis from pulmonary adenosquamous carcinoma is even rarer, which up until now has only been reported once in the literature, with our case being the second [3].

MR imaging is a particularly advantageous imaging modality in evaluating secondary penile metastases due to the superior soft tissue resolution, multiplanar functionality, and ability to accurately characterize disease extension [15]. In the setting of lung cancer, penile metastases most commonly involve the corpora cavernosa in the shaft with one meta-analysis reporting 85% of lesions in this location [3, 6, 10]. As was demonstrated in our case, both cavernosa are involved in the majority of cases which may be attributed to free communication through an incomplete intercorporal septum [3]. Penile metastases generally show hypointensity on T1-weighted imaging with a variable appearance on T2-weighted imaging ranging from hypointense to slightly hyperintense compared to the cavernosa [10, 15]. Following administration of intravenous contrast, metastatic lesions typically avidly enhance [10, 15]. In lesions with increased metabolic activity, central areas of hypoenhancement may be present, reflecting areas of central necrosis, as was demonstrated in our case. In addition to a discrete mass, an infiltrative appearance has also been described [15].

PET/CT has been described as a valuable imaging tool in the setting of secondary penile metastases for detection of the primary malignancy, identification of additional sites of metastases, and the facilitation of staging, which may all be provided in a single examination [3, 16, 17]. Moreover, in asymptomatic patients, PET/CT may incidentally detect small penile metastases that would otherwise be difficult to assess on conventional CT imaging or physical examination [17]. Secondary penile metastases demonstrate similar metabolic activity to the primary neoplasm, as was seen in our case, where the penile metastases, primary lung lesion, adrenal lesion, and right subscapularis lesion all demonstrated similar hypermetabolic activity. To the best of our knowledge, there has been no study investigating SUV_max_ as a measure of distinguishing a primary penile malignancy versus metastases from a synchronous neoplasm; however, a meta-analysis may be difficult given the rarity of cases. In the posttreatment setting, PET/CT is particularly advantageous for disease restaging when assessing for treatment modification [16, 17].

Choice of treatment in the setting of metastatic penile cancer is multifactorial and is based on the type of cancer, size and number of metastatic lesions, age, and patient constitution [3, 6]. With the vast majority of patients with penile metastases presenting with widespread metastatic disease and a mean survival time of 4.5 to 5.5 months, treatment is usually palliative, involving local resection or radiation therapy to improve the overall quality of life [3, 6, 18]. Additional treatment strategies include chemotherapy as was chosen by the patient in our case [3, 6]. In the setting of urinary obstruction, suprapubic catheterization may be performed and in cases of a large mass producing intractable pain, penectomy has been reported [6].

In conclusion, this is a case of secondary penile metastases as the initial presentation of metastatic pulmonary ASC and is, to the best of our knowledge, only the second described case of this entity. Penile metastases are rarely identified prior to the diagnosis of the primary neoplasm. Given the rarity of secondary penile metastases from primary lung cancer and the poor prognosis following identification, knowledge of this atypical heralding lesion to prompt further assessment for end-stage extrapelvic metastatic malignancy is crucial when evaluating these patients.
